# Selective Neural Deletion of the *Atg7* Gene Reduces Irradiation-Induced Cerebellar White Matter Injury in the Juvenile Mouse Brain by Ameliorating Oligodendrocyte Progenitor Cell Loss

**DOI:** 10.3389/fncel.2019.00241

**Published:** 2019-05-31

**Authors:** Yafeng Wang, Kai Zhou, Tao Li, Yiran Xu, Cuicui Xie, Yanyan Sun, Juan Rodriguez, Shan Zhang, Juan Song, Xiaoyang Wang, Klas Blomgren, Changlian Zhu

**Affiliations:** ^1^Henan Key Laboratory of Child Brain Injury, Third Affiliated Hospital and Institute of Neuroscience, Zhengzhou University, Zhengzhou, China; ^2^Center for Brain Repair and Rehabilitation, Institute of Neuroscience and Physiology, Sahlgrenska Academy, University of Gothenburg, Gothenburg, Sweden; ^3^Department of Pediatrics, Children’s Hospital Affiliated to Zhengzhou University, Zhengzhou, China; ^4^Department of Women’s and Children’s Health, Karolinska Institutet, Stockholm, Sweden; ^5^Perinatal Center, Institute of Neuroscience and Physiology, Sahlgrenska Academy, University of Gothenburg, Gothenburg, Sweden; ^6^Pediatric Oncology, Karolinska University Hospital, Stockholm, Sweden

**Keywords:** autophagy, cell proliferation, inflammation, microglia, oligodendrocyte progenitor cells, astrocyte, white matter injury

## Abstract

Radiotherapy is an effective tool for treating brain tumors, but irradiation-induced toxicity to the normal brain tissue remains a major problem. Here, we investigated if selective neural *autophagy related gene 7* (*Atg7*) deletion has a persistent effect on irradiation-induced juvenile mouse brain injury. Ten-day-old *Atg7* knockout under a nestin promoter (KO) mice and wild-type (WT) littermates were subjected to a single dose of 6 Gy whole-brain irradiation. Cerebellar volume, cell proliferation, microglia activation, inflammation, and myelination were evaluated in the cerebellum at 5 days after irradiation. We found that neural *Atg7* deficiency partially prevented myelin disruption compared to the WT mice after irradiation, as indicated by myelin basic protein staining. Irradiation induced oligodendrocyte progenitor cell (OPC) loss in the white matter of the cerebellum, and *Atg7* deficiency partly prevented this. The mRNA expression of oligodendrocyte and myelination-related genes (*Olig2*, *Cldn11*, *CNP*, and *MBP*) was higher in the cerebellum in *Atg7* KO mice compared with WT littermates. The total cerebellar volume was significantly reduced after irradiation in both *Atg7* KO and WT mice. *Atg7*-deficient cerebellums were in a regenerative state before irradiation, as judged by the increased OPC-related and neurogenesis-related transcripts and the increased numbers of microglia; however, except for the OPC parameters these were the same in both genotypes after irradiation. Finally, there was no significant change in the number of astrocytes in the cerebellum after irradiation. These results suggest that selective neural *Atg7* deficiency reduces irradiation-induced cerebellar white matter injury in the juvenile mouse brain, secondary to prevention of OPC loss.

## Introduction

Brain tumors are the second most common childhood malignancies. Excellent survival rates have been achieved for some types of brain tumors using multimodal treatment approaches that contain radiotherapy as an integral component. However, adverse reactions to irradiation in the surrounding normal brain tissue remain a major problem (Kalapurakal et al., 2006; Di Giannatale et al., 2014). The immature infant brain is still developing and is more sensitive than the mature adult brain to the negative side effects of irradiation (Blomstrand et al., 2014). Cognitive impairments, metabolic and endocrine disturbances, secondary malignant tumors, delayed growth, and other long-term effects have been seen in children after radiotherapy (Piccardo et al., 2012; Laprie et al., 2015; Xu et al., 2018). Thus, preventing long-term irradiation-induced sequelae is a crucial concern for improving cancer survivors’ quality of life (Naylor et al., 2008; Huo et al., 2012; Zhou et al., 2017b).

Due to improvements in radiotherapy treatment techniques, the more pediatric patients are surviving their brain tumors, but this has also led to a greater number of children suffering from the late-onset consequences of radiotherapy (Rodgers et al., 2013; Ibrahim et al., 2014), especially in terms of cognitive deficits (Balentova and Adamkov, 2015). High-energy radiotherapy has long been implicated in DNA damage and cell death, and late-onset radiation-induced changes are often progressive and irreversible. Such therapy often leads to diffuse white matter changes ranging from scattered focal white matter lesions to confluent lesions, and the type of lesion varies greatly, including mineralizing microangiopathy, diffuse cerebral atrophy, focal areas of frank radiation necrosis, and radiation-induced vasculopathy (Dietrich et al., 2001; Fouladi et al., 2004; Kralik et al., 2015, 2017; Szychot et al., 2017).

Autophagy is crucial for cell survival, differentiation, development, and homeostasis (Oppenheim et al., 2008; Xie et al., 2014; Huang et al., 2018; Zhao et al., 2018), but although autophagy plays a role in the removal of damaged and harmful components in cells, inappropriate activation of autophagy in the immature brain is associated with cell death and with childhood neurological disorders (Descloux et al., 2015; Lei et al., 2017; Makale et al., 2017; Wang et al., 2017). In two of our recent studies, we showed that genetic inhibition of autophagy was neuroprotective in the juvenile mouse brain in the acute injury stage (Xie et al., 2016; Wang et al., 2017), but it was still unknown if the inhibition of autophagy has persistent effects in the subacute phase after insult to the juvenile mouse brain (2–7 days after cerebral irradiation when most of the radiation-induced cell death has disappeared).

The aim of this study was to determine if selective neural inhibition of *autophagy related gene 7* (*Atg7*) in the juvenile mouse brain has persistent effects after cerebral irradiation. We found that selective neural autophagy inhibition reduced irradiation-induced white matter injury in the cerebellum in the subacute phase and that this probably was related to reduced OPC death.

## Materials and Methods

### Animals and Ethical Permission

*Atg7*^*flox/flox*; Nes–Cre^ knockout (*Atg7* KO) and *Atg7*^*flox*/+; Nes–Cre^ mice (WT) and their genotyping were generated by crossbreeding floxed *Atg7* mice and nestin-Cre-driven mice as described in our previous work (Xie et al., 2016; Wang et al., 2017). The mice were housed in a 12:12-h light/dark cycle with food and water freely available. All experiments were approved by the animal research ethics committee (Gothenburg Committee of the Swedish Agricultural Agency) in accordance with national animal welfare legislation (112-2014).

### Irradiation Procedure

On postnatal day 10 (P10), littermate pups of both sexes in the *Atg7* KO and WT groups were anesthetized with a 50 mg/kg intraperitoneal injection of tribromoethanol (Avertin, Sigma-Aldrich, Stockholm, Sweden). Animals were put in a prone position (head to gantry) on a Styrofoam bed. A linear accelerator (Varian Clinic 600CD; Radiation Oncology System LLC, San Diego, CA, United States) with 4 MV nominal photon energy and a dose rate of 2.3 Gy/min was used to the irradiate the mice. A single dose of 6 Gy was given to the whole brain of each mouse. This represents a clinically relevant low to moderate radiation dose. The source-to-skin distance was 99.5 cm. In order to obtain an even irradiation dose throughout the tissue, the head was covered with a 1 cm tissue-equivalent bolus material. After the pups were irradiated, they were returned to their dams and sacrificed at 5 days after irradiation. The mice in the sham-irradiated control group were anesthetized but not subjected to irradiation.

### Immunohistochemistry Staining

At 5 days after irradiation, the animals were deeply anesthetized with an overdose of sodium phenobarbital and perfused intracardially with PBS and 5% buffered formaldehyde (Histofix; Histolab, Gothenburg, Sweden). The animals’ brains were removed and fixed in 5% buffered formaldehyde at 4°C for 24 h, followed by dehydration with graded ethanol and xylene. The brains were paraffin-embedded and cut into 5 μm sagittal sections. Every 50th section from one hemisphere was used for active Ki-67, PDGFRα, MBP, Iba-1, and GFAP staining. After deparaffinization with xylene and ethanol and antigen recovery, the sections were blocked for 30 min with 4% donkey serum (for MBP staining, 4% donkey serum with 0.2% Triton X-100 and 3% bovine serum albumin) in PBS for 30 min. The monoclonal rabbit anti-Ki-67 (1:400 dilution, Abcam, ab15580), rabbit anti-Iba-1 (1:200 dilution, Wako Pure Chemical Industries, Ltd., 019-19741), mouse anti-MBP (1:1,000 dilution, BioLegend, SMI 94, 836504), rabbit anti-PDGFRα (1:400 dilution, Cell Signaling, 3164), and mouse anti-GFAP (1:250 dilution, Cell Signaling, 3670) primary antibodies were incubated overnight at 4°C. The appropriate biotinylated secondary antibodies (1:200 dilution for Ki-67, Iba-1, PDGFRα, and GFAP staining; 1:250 dilution for MBP staining; all from Vector Laboratories, Burlingame, CA, United States) were added and incubated for 60 min at room temperature. After blocking endogenous peroxidase activity with 3% H_2_O_2_ for 10 min, visualization was performed by using Vectastain ABC Elite (Vector Laboratories) with 3,3′-diaminobenzidine. After dehydrating with graded ethanol and xylene, the sections were mounted using Vector mounting medium.

### Cell Quantification, Volume Measurement, and White Matter Injury Evaluation in Mice

Ki-67, Iba-1, PDGFRα, and GFAP-positive cells were counted in the cerebellum using stereology microscopy (MicroBrightField, Magdeburg, Germany). The counting areas in the EGL, ML, cerebellar IGL, and cerebellar white matter were traced in the cerebellum, and the number of cells was expressed as cells/mm^2^ (Wang et al., 2017). As described in our previous work, the second cerebellar lobule was selected for counting of Ki-67 staining while the cerebellar white matter was selected for counting of PDGFRα and GFAP staining. Iba-1 was counted in the whole section. According to the morphology classification, the Iba-1–labeled cells were classified into ramified (surveillance phenotype/non-activated: characterized by long, ramified processes with comparatively small cell bodies), hyper-ramified (reactive/intermediate: characterized by thicker primary processes and retracting secondary processes), or un-ramified (activated), including bushy (characterized by swollen, truncated processes, and enlarged cell bodies) or amoeboid (characterized by rounded macrophage-like morphology with no or few processes) microglia (Xie et al., 2014; Wang et al., 2017). Regional volumes were calculated according to the Cavalieri principle using the formula described previously (Sun et al., 2016; Wang et al., 2017). For measuring the volume of the cerebellum, brain sections were stained by hematoxylin and eosin staining. The cerebellar volume and cerebellar MBP-positive white matter volume (mm^3^) were calculated as previously described (Sun et al., 2016; Zhang et al., 2017) using the following formula: *V* = *S*_A_ × *p* × *T*, where *V* is the total volume, *S*_A_ is the sum of the areas measured, *p* is the inverse of the section sampling fraction, and *T* is the section thickness. MBP-positive area and MBP-positive immunodensity were determined by using ImageJ software as described previously (Zhang et al., 2017).

### RNA Isolation and cDNA Synthesis

Total RNA was isolated using the RNeasy mini kit (Qiagen, 74104) according to the manufacturer’s instructions. The concentration and purity of all RNA samples were determined using a NanoDrop spectrophotometer (NanoDrop Technologies, Wilmington, DE, United States). The integrity of the RNA was measured using the Experion RNA StdSens analysis kit (Bio-Rad, 7007103) on an Automated Electrophoresis Station (Bio-Rad, Hercules, CA, United States). One microgram of total RNA was reverse transcribed using the QuantiTect Reverse Transcription kit (Qiagen, 205311).

### Quantitative Real Time PCR

Quantitative real time PCR (qRT-PCR) was performed using a LightCycler 480 instrument (Roche Diagnostics, Germany) and the SYBR Green technique according to the manufacturer’s instructions. The primers used in the qRT-PCR reactions were designed by Beacon Designer software (free trial, PREMIER Biosoft) and included the stem cell and proliferation genes *Ki-67* (sense: 5′-GCC TCC TAA TAC ACC ACT GA-3′, antisense: 5′-CCG TTC CTT GAT GAT TGT CTT-3′), *DCX* (sense: 5′-GAC AAC ATT AAC CTG CCT CA-3′, antisense: 5′-CCT TCT TCC AGT TCA TCC AT-3′), and *SOX2* (sense: 5′- CGC AGA CCT ACA TGA ACG-3′, antisense: 5′-CTC GGA CTT GAC CAC AGA-3′); the oligodendrocyte and myelin-related genes *Olig2* (sense: 5′-CGG TGG CTT CAA GTC ATC-3′, antisense: 5′-GTC ATC TGC TTC TTG TCT TTC T-3′), *Cldn11* (sense: 5′-TGG CAT CAT CGT CAC AAC-3′, antisense: 5′-AGC CCA GTT CGT CCA TTT-3′), *CNP* (sense: 5′-TCT ACT TTG GCT GGT TCC T-3′, antisense: 5′-CTT CTC CTT GGG TTC ATC TC-3′), and *MBP* (sense: 5′-CCT CAC AGC GAT CCA AGT-3′, antisense: 5′-CAA GGA TGC CCG TGT CTC-3′); and the astrocyte-related genes *GFAP* (sense: 5′-GAG GTG GAG AGG GAC AAC-3′, antisense: 5′-TCT ATA CGC AGC CAG GTT-3′) and *Vimentin* (sense: 5′-TTC AAG ACT CGG TGG ACT-3′, antisense: 5′-GCA GTT CTA CCT TCT CGT T-3′). The reference gene was *Sdha* (sense: 5′-TTG CCT TGC CAG GAC TTA-3′, antisense: 5′-CAC CTT GAC TGT TGA TGA GAA T-3′). The relative expression levels of mRNAs were calculated according to the formula of 2^−(ΔΔCT)^.

### Multiplex Cytokine/Chemokine Assay

Cytokines and chemokines were measured in cerebellum homogenate supernatant fractions at 5 days after irradiation. Protein concentrations were measured with the BCA protein assay (Sigma, A2058) using samples prepared according to the manufacturer’s protocol. Levels of IL-1β, IL-2, IL-4, IL-6, IL-10, KC, and CCL2 were measured simultaneously using the Luminex Multiplex Cytokine Assay (Merck Chemicals and Life Science AB). The results were normalized to the amount of protein in the sample.

### Statistical Analysis

We used the Statistical Package for the Social Sciences 17.0 (SPSS, IBM, NY, United States) to analyze all of the data. Student’s *t*-test was used for the comparisons between groups, and the Mann–Whitney *U*-test was used for comparison of the unequal variance data. Multiple comparisons with data from more than two groups were performed by two-way ANOVA followed by a Bonferroni post hoc test. The results are presented as means ± SEM, and *p* < 0.05 was considered a significant difference.

## Results

### Irradiation Induced Cerebellar White Matter Injury

**FIGURE 1:**
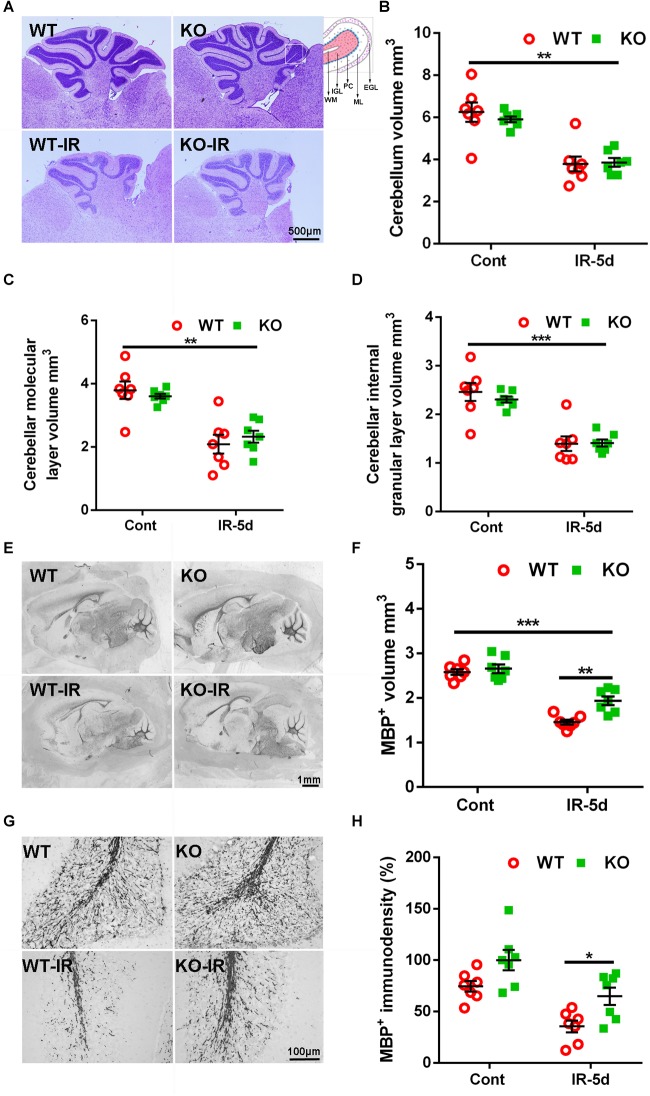
Cerebral irradiation-induced cerebellar white matter injury. **(A)** Representative hematoxylin and eosin staining in the cerebellum. Each folia comprises distinct cellular layers, including the external germinal layer (EGL); molecular layer (ML); Purkinje cell layer (PC), internal granular layer (IGL), and white matter (WM). **(B)** The cerebellar volume was significantly smaller at 5 days after irradiation compared to the non-irradiated control group. **(C)** The volume of the cerebellar ML. **(D)** The volume of the cerebellar IGL. **(E)** Representative MBP staining in the sagittal brain sections at 5 days after irradiation. **(F)** *Atg7* KO reduced the loss of MBP-positive volume after irradiation. **(G)** Representative MBP immunostaining in white matter from cerebellar lobules. **(H)** *Atg7* KO prevented the loss of MBP-positive immunodensity after irradiation. *n* = 7/group. ^∗^*p* < 0.05, ^∗∗^*p* < 0.01, ^∗∗∗^*p* < 0.001.

Cerebral irradiation induces cell death in proliferating cells, peaking after 6 h, and this cell death decreases dramatically by 24 h after irradiation (Zhou et al., 2017a). In accordance with this, we observed very few pyknotic cells in the cerebellum at 5 days after irradiation (Figure 1A). The cerebellar volume was significantly smaller after irradiation [6.25 ± 0.45 mm^3^ (WT un-irradiated) vs. 3.79 ± 0.42 mm^3^ (WT irradiated); 5.91 ± 0.16 mm^3^ (*Atg7* KO un-irradiated) vs. 3.86 ± 0.24 mm^3^ (*Atg7* KO irradiated), *p* < 0.01], but the differences between WT and *Atg7* KO mice were not significant (Figure 1A,B). Similar reductions were observed in the cerebellar ML (*p* < 0.01) and the IGL (*p* < 0.001) at 5 days after irradiation (Figure 1C,D).

Myelination was visualized in the cerebellum as indicated by MBP staining (Figure 1E). The MBP-positive volume decreased significantly at 5 days after irradiation in the cerebellar white matter (*p* < 0.001), but the volume of the cerebellar white matter was greater in *Atg7* KO pups compared to WT littermates after irradiation (1.94 ± 0.11 mm^3^ vs. 1.46 ± 0.06 mm^3^, respectively, *p* = 0.001) (Figure 1F). Further analysis of the MBP-positive immunodensity in the cerebellar white matter (Figure 1G) showed that *Atg7* deficiency caused less obvious myelin disruption compared to the WT littermates after irradiation (*p* = 0.023), while no difference was seen between WT and KO pups under physiological conditions (*p* = 0.052) (Figure 1H).

### *Atg7* Deficiency Reduced Irradiation-Induced Cerebellar OPC Loss

**FIGURE 2:**
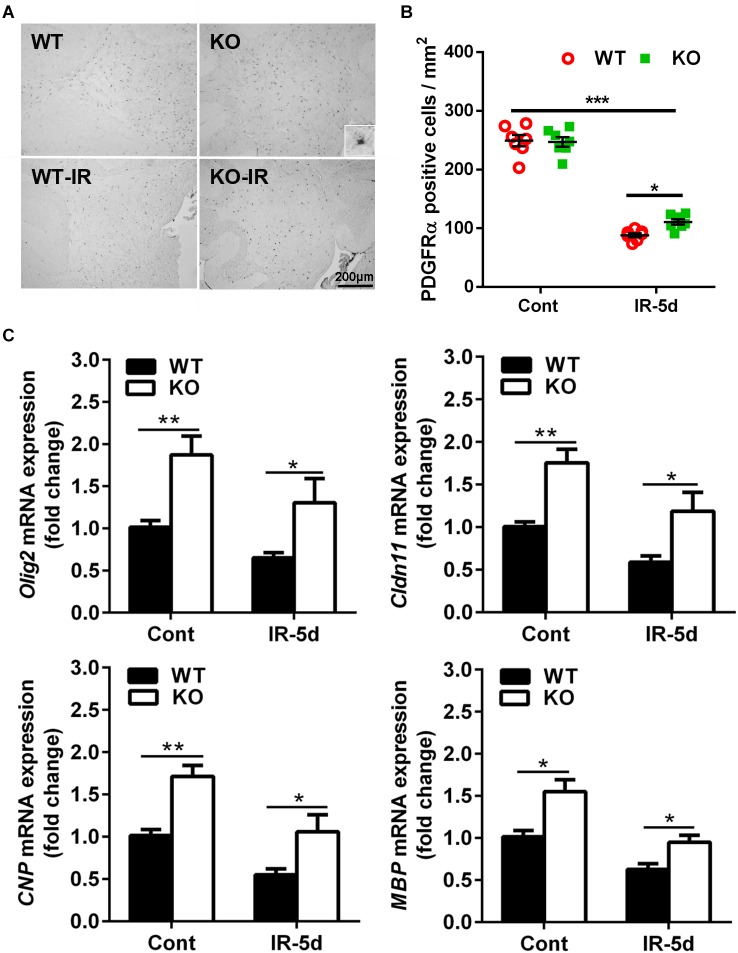
*Atg7* KO reduced irradiation-induced OPC loss. **(A)** Mouse OPCs in the cerebellum after irradiation were immunostained for PDGFRα. **(B)** Quantitative analysis of PDGFRα-labeled cells in the cerebellar white matter. **(C)** The mRNA level of *Olig2*, *Cldn11*, *CNP*, and *MBP* was significantly greater in the *Atg7* KO mice compared with WT mice under both physiological conditions and after irradiation. *n* = 7/group for immunohistochemistry staining; *n* = 5/group for the qRT-PCR assays. ^∗^*p* < 0.05, ^∗∗^*p* < 0.01, ^∗∗∗^*p* < 0.001.

To determine the extent of irradiation-induced white matter injury, we examined the influence of irradiation on OPCs. PDGFRα is a marker of OPCs (Yeh et al., 1993), and PDGFRα-positive cells were located mainly in the cerebellar white matter (Figure 2A). The numbers of PDGFRα-positive cells were much lower in both groups of mice after irradiation (*p* < 0.001); however, there were more PDGFRα-positive cells in the *Atg7* KO mice compared with WT mice at 5 days after irradiation (110.8 ± 4.58 cells/mm^2^ vs. 88.2 ± 3.67 cells/mm^2^, respectively, *p* = 0.033) (Figure 2B).

We then measured the mRNA expression of oligodendrocyte-related and myelin-related genes in the cerebellum (Figure 2C). *Olig2*, *Cldn11*, *CNP*, and *MBP* mRNA expression was significantly greater in the *Atg7* KO mice compared with WT mice under both physiological conditions and after irradiation, and irradiation reduced the expression of these genes (*Olig2*: reduced by 35.6% in WT mice and by 30.5% in *Atg7* KO mice after irradiation; *Cldn11*: reduced by 41.0% in WT mice and by 32.3% in *Atg7* KO mice after irradiation; *CNP*: reduced by 45.5% in WT mice and by 38.0% in *Atg7* KO mice after irradiation: *MBP*: reduced by 38.0% in WT mice and by 38.7% in *Atg7* KO mice after irradiation).

### Irradiation Reduced Neural Stem/Progenitor Cell Proliferation in the Cerebellum

**FIGURE 3:**
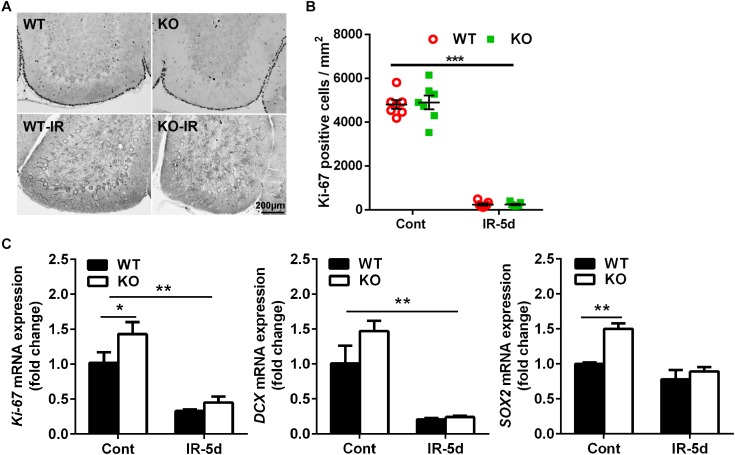
Irradiation reduced neural stem/progenitor cell proliferation in the cerebellum. **(A)** Representative pictures showing Ki-67-labeled cells in the cerebellum. **(B)** Quantitative analysis of Ki-67–labeled cells in the EGL of the cerebellar lobule. **(C)** Bar graphs showing mRNA level of *Ki-67*, *DCX*, and *SOX2* in the cerebellum. *n* = 7/group for immunohistochemistry staining; *n* = 5/group for the qRT-PCR assays. ^∗^*p* < 0.05, ^∗∗^*p* < 0.01, ^∗∗∗^*p* < 0.001.

To investigate the impact of neural inhibition of *Atg7* on neural stem and progenitor cell proliferation, we measured Ki-67 labeling (Figure 3A,B) and mRNA expression (Figure 3C) in the cerebellum. Ki-67–labeled cells were mainly located in the EGL (Figure 3A), and quantification of Ki-67–labeled cells showed a massive reduction in the EGL of the cerebellum after irradiation; however, there was no difference between *Atg7* KO and WT littermates under physiological conditions or at 5 days after irradiation (Figure 3B). The expression of *Ki-67* and *DCX* mRNA decreased significantly after irradiation compared to non-irradiated littermates (*p* < 0.01), but no differences were seen between *Atg7* KO and WT littermates after irradiation. However, the expression of *Ki-67*, *SOX2*, and *DCX* mRNA was obviously elevated in *Atg7* KO mice compared to WT in the cerebellum under physiological conditions (Figure 3C).

### Microglia Activation in the Cerebellum After Irradiation

**FIGURE 4:**
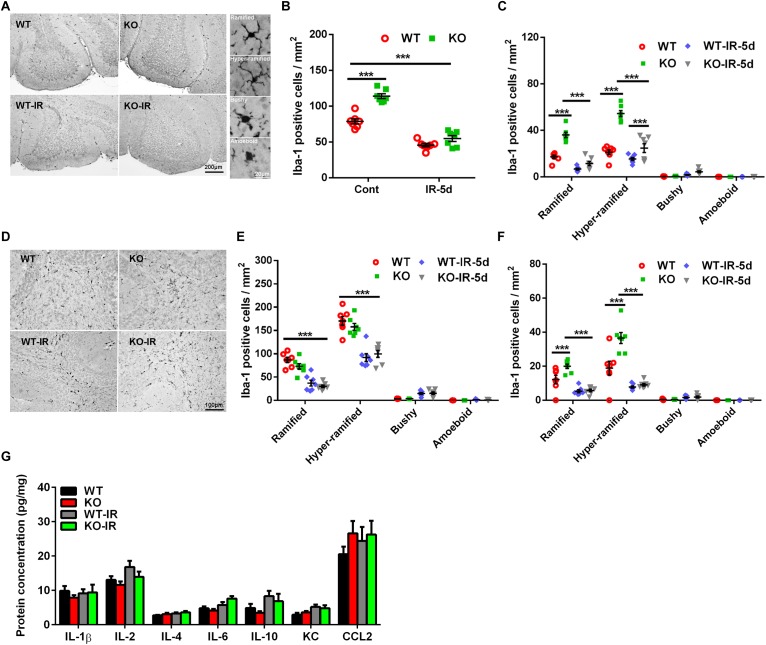
Morphological changes in microglia in the cerebellum after irradiation. **(A)** Microglia in the cerebellum were immunostained by Iba-1 and classified into ramified, hyper-ramified, bushy, and amoeboid phenotypes based on their morphological characteristics. **(B)** The number of Iba-1–labeled cells in the cerebellum decreased significantly at 5 days after irradiation. **(C)** Iba-1–labeled cells with different morphologies were counted separately in the cerebellar IGL. **(D)** Representative pictures showing the Iba-1–labeled cells in the cerebellar white matter. **(E)** Iba-1–labeled cells with different morphologies were counted separately in white matter of the cerebellum. **(F)** Iba-1–labeled cells with different morphologies were counted separately in the cerebellar ML. **(G)** The protein levels of IL-1β, IL-2, IL-4, IL-6, IL-10, KC, and CCL2 in the cerebellum were detected by Luminex assay after irradiation in the *Atg7* KO and WT pups. *n* = 7/group for the Iba-1 staining and Luminex assay. ^∗∗∗^*p* < 0.001.

Our previous study demonstrated that irradiation could induce microglia activation and inflammation in the neurogenic regions, and neural autophagy deficiency decreased microglia activation after irradiation in the acute phase in the juvenile mouse brain, but the effect of autophagy deficiency on irradiation-induced subacute brain injury was still not clear (Monje et al., 2003; Han et al., 2016; Wang et al., 2017). In the present work, as indicated by Iba-1 immunochemistry staining, microglia were scattered throughout the normal brain and became un-ramified, or activated (bushy or amoeboid), after irradiation (Figure 4A). The number of microglia was greater in the *Atg7* KO group under physiological conditions compared with WT mice (*p* < 0.001), and the number of microglia in the cerebellum decreased significantly at 5 days after irradiation (*p* < 0.001) but with no significant difference between the *Atg7* KO and WT groups (Figure 4B). Microglia with different morphologies were counted separately in the cerebellar IGL (Figure 4C), white matter (Figure 4D,E), and ML (Figure 4F). There were fewer non-activated (ramified and hyper-ramified) microglia in the IGL, ML, and white matter after irradiation (*p* < 0.001) (Figure 4C,E,F). We also measured the protein levels of IL-1β, IL-2, IL-4, IL-6, IL-10, KC, and CCL2 in the cerebellum and found no difference at 5 days after irradiation between the *Atg7* KO and WT groups (Figure 4G).

### Astrocyte Reactivity in the Cerebellum After Irradiation

**FIGURE 5:**
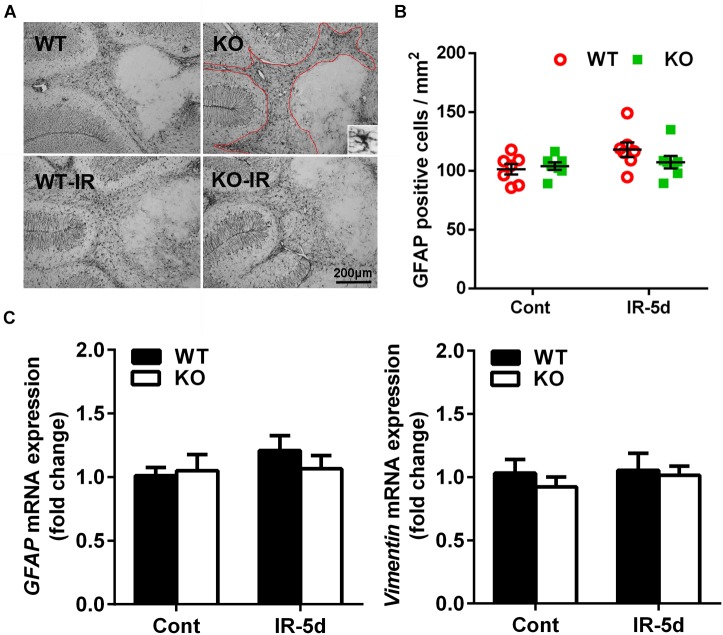
Astrocyte reactivity in the cerebellum after irradiation. **(A)** Representative pictures showing GFAP-labeled cells in the cerebellum. **(B)** Quantitative analysis of GFAP-labeled cells in cerebellar white matter. **(C)** The mRNA expression of *GFAP* and *Vimentin* in the cerebellum. *n* = 7/group for GFAP staining; *n* = 5/group for the qRT-PCR assays.

Astrocytes, as indicated by GFAP labeling, were detected throughout the cerebellum (Figure 5A), and the numbers of GFAP-labeled cells did not show statistical difference in the cerebellum between the non-irradiated and irradiated mice or between *Atg7* KO and WT mice (Figure 5B). We then measured the expression of astrocyte-related genes and found no differences in the expression of *GFAP* or *Vimentin* between the *Atg7* KO and WT groups under physiological conditions or after irradiation, which indicated that irradiation did not influence the mRNA expression of *GFAP* or *Vimentin* in the cerebellum (Figure 5C).

## Discussion

Autophagy is an evolutionarily highly conserved, lysosome-dependent cellular recycling pathway in eukaryotic cells that recycles cytoplasmic components and plays a critical role in cell adaptation and in the clearance of intra-cellular pathogens, and it prevents aging and tumor development (Hao et al., 2018; Pellacani and Costa, 2018). The *Atg7* gene plays a critical role in the process of autophagy (Park et al., 2016), and selective neural *Atg7* deficiency demonstrates that neuronal survival requires autophagy at basal levels (Oppenheim et al., 2008). However, over-activation of autophagy induces neural cell injury, and autophagy inhibition prevents stress-induced neural cell death (Xie et al., 2016; Bisicchia et al., 2017). We recently demonstrated that selective *Atg7* deletion decreases neural stem and progenitor cell death after irradiation and reduces irradiation-induced caspase-3 activation, microglia activation, and inflammation during the acute injury phase (Wang et al., 2017). But, it was still not clear if autophagy deficiency could prevent irradiation-induced subacute brain injury in the juvenile brain. In the current study, we show that selective neural autophagy deletion reduces irradiation-induced cerebellar white matter injury by decreasing OPC loss.

Cranial irradiation, even a single moderate dose, leads to massive neural stem cell death (Fukuda et al., 2004) followed by changes in cell metabolism, the cellular microenvironment, cell proliferation, and tissue shape as well as long-term cognitive impairments and growth reduction (Sydoruk et al., 2014; Walenta and Mueller-Klieser, 2016). Our previous results showed that selective *Atg7* deficiency reduced neural stem cell death at 6 h after irradiation. In this study, cell death was barely detected in the cerebellum at 5 days after irradiation, but there was dramatically decreased cerebellar volume as a result of reduced growth. These results indicate that irradiation-induced neural stem/progenitor cell death occurs in the early stages of radiation-induced injury but is no longer present at 5 days after irradiation, and taken together these studies show that autophagy inhibition merely delays, rather than prevents, neural stem/progenitor cell death.

Irradiation of the rodent brain has been shown to result in reduced oligodendrocyte numbers and myelin loss (Bull et al., 2017), more so in females (Roughton et al., 2013), and a previous rodent study showed that white matter development in the brain was inhibited after irradiation on postnatal day 8, resulting in a 50–70% reduction in MBP staining (Fukuda et al., 2004). In this study, the MBP-positive volume and immunodensity were greater in the cerebellar white matter of *Atg7* KO mice compared to WT control littermates after irradiation, indicating that neural autophagy inhibition reduced the severity of irradiation-induced cerebellar white matter injury in the juvenile mouse brain.

Autophagy involves a decline in the number and functionality of stem cells/progenitor cells (Revuelta and Matheu, 2017). Under pathological conditions such as oxidative stress, starvation, hypoxia, or irradiation, autophagy is enhanced in order to eliminate cell debris and damaged organelles, but over-activation of autophagy can also induce cell death and tissue injury (Ravanan et al., 2017). A great number of OPCs residing in the white matter are eliminated by exposure to irradiation during the peak of gliogenesis (Greene-Schloesser et al., 2012; Burns et al., 2016). Premitotic apoptosis is a rapid process of cell death that follows high-intensity radiation and is characterized by rapid activation of caspase-3, while postmitotic apoptosis is a delayed process of cell death following cell division (Shinomiya, 2001). Morphologically, OPCs have small cell bodies and multiple branched processes (Wang and Almazan, 2016), and they also have synaptic junctions with neurons (Bergles et al., 2000) and are thus involved in the formation of myelinated axons (Pang et al., 2018). In a conditional knockout mouse model, deletion of *Atg7* signaling in neurons resulted in significant and long-lasting resistance of their axons to retrograde degeneration, which was observed as an increased number of preserved axons and striatal dopaminergic nerve terminals at 4 weeks after brain injury (Cheng et al., 2011). Another study demonstrated that the autophagy inhibitor 3-methyladenine could clearly delay axonal degeneration and stabilize the degenerating axons following acute neuron injury (Knoferle et al., 2010). In our juvenile mouse model, *Atg7* deficiency reduced OPC loss in the cerebellar white matter. We also measured the expression of the oligodendrocyte-related genes *Olig2*, *Cldn11*, and *CNP* and the myelin-related gene *MBP*, and our results strongly suggest that neural *Atg7* KO promotes oligodendrocyte differentiation and maturation. Taken together, our results indicate that deficiency in neural autophagy prevents, or at least delays, radiation-induced white matter injury by preventing OPC loss, and this probably involves delayed axonal degeneration. Thus further research is needed to investigate if delayed axonal degeneration alone prevents OPC death or if there are additional mechanisms explaining how neural autophagy deficiency can protect OPCs.

Under physiological conditions, autophagy plays essential roles in the regulation of cell differentiation and proliferation (Sanchez-Vera et al., 2017; Wu et al., 2017; Chen et al., 2018). The present data showed that the numbers of Ki-67-labeled cells in the cerebellum were drastically lower in the irradiated mice compared to the non-irradiated mice in the subacute phase and were much lower than in the acute phase at 6 h after irradiation (Wang et al., 2017), which suggests that neural stem cell numbers do not recover after a moderate dose of irradiation, and rather the opposite is seen analogous to our observations in the hippocampus (Bostrom et al., 2013). Autophagy inhibition had no influence on cell proliferation or neurogenesis in the cerebellum despite the fact that it caused increased stem cell proliferation-related and neurogenesis-related gene expression under physiological conditions. This indicates that neural autophagy deficiency is stressful to the tissue and causes a state of regeneration in the *Atg7*-deficient cerebellum. Combined with the results of cell death at the acute phase from our previous study (Wang et al., 2017), the greater concentrations of neurogenesis-related transcription markers indicates that the loss was relatively greater in the *Atg7* KO brains and was at the same level as in WT brains. This might be interpreted as blunting, or even ablating, the regenerative response after irradiation.

As the resident mononuclear phagocytes in the brain, microglia maintain homeostasis of the micro-environment and associate with immune defense. They are involved in the pathophysiology of neurodegenerative diseases (Stranks et al., 2015; Bisicchia et al., 2017; De Picker et al., 2017; Plaza-Zabala et al., 2017) and become activated after cerebral insults. Microglia activation is characterized by rounding of the cell body, retraction of cell processes, proliferation, and elevated expression of both pro- and anti-inflammatory cytokines and chemokines (Iwahara et al., 2017; Chi et al., 2018). We previously showed that irradiation induces microglia activation and that these cells accumulate in the injured area in the acute phase (Wang et al., 2017). In the present work, the total number of microglia was significantly reduced at 5 days after irradiation compared to the non-irradiated controls, and only very few activated microglia were seen in the cerebellum at this time point. It is conceivable that activated microglia are more susceptible to irradiation-induced death (Han et al., 2016), and the higher numbers of microglia in *Atg7*-deficient brains could then explain the relatively greater loss of microglia in the cerebellum. Analogous to the changes in microglia numbers and reactivity from the acute to the subacute phase, inflammation, as indicated by cytokines and chemokine levels in the cerebellum, was not significantly different between irradiated and non-irradiated mice or between *Atg7* KO and WT control littermates at this time point. Interestingly, *Atg7* deletion promoted microglia accumulation in the cerebellum under physiological conditions, probably related to *Atg7* deficiency-induced chronic neurodegeneration.

In addition to microglia, astrocytes are also related to chronic inflammation in the brain (Makale et al., 2017; Xu et al., 2018). Astrocyte reactivity can induce inflammation and reduce the integrity of the BBB, which can allow cytokines and chemokines to enter the brain from the periphery (Fuente-Martin et al., 2013; Shiow et al., 2017). Irradiation can disrupt the neurovascular niche in the juvenile developing brain (Bostrom et al., 2014; Zhou et al., 2017a), and impaired BBB integrity is involved in increased levels of MMP-9, which is one of the MMP family proteins that is secreted by the neuroglia and is involved in sensitivity to neuro-inflammation (Hernandez-Guillamon et al., 2009; Li et al., 2013). However, clear disruption of BBB integrity and a significant increase in MMP-9 expression only occurred in the late phase (4–8 weeks) after irradiation (Li et al., 2013; Sandor et al., 2014). Thus, the methodology in the current study investigating brain injury in the subacute phase was not suitable for analyzing BBB integrity. As for astrocyte reactivity, we found that there were no significant changes in the numbers of astrocytes or in the expression of astrocyte-related genes in the cerebellum between irradiated and non-irradiated mice or between *Atg7* KO and WT mice. This suggests that astrocytes do not play a key role in irradiation-induced cerebellar white-matter injury.

**FIGURE 6:**
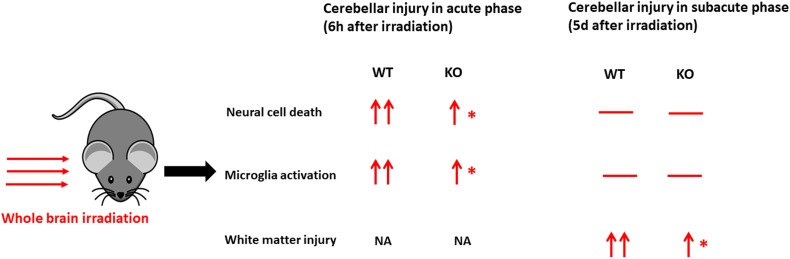
A schematic illustration of comparison in cerebellar injury between the acute phase and subacute phase after a single moderate dose of irradiation. Irradiation induces cell death, microglial activation, and white matter injury in the cerebellum at different time points. —, no significant change; ↑, significant increase compared with the control group without irradiation (vs. cont); ↑↑, a more significant increase (vs. cont); ^∗^significant difference compared with WT after irradiation (*p* < 0.05). WT, wild type; KO, knock out; NA, not available.

In conclusion, compared with the acute phase after irradiation in our previous work (Wang et al., 2017) (Figure 6), selective neural *Atg7* KO reduced irradiation-induced cerebellar white matter injury in the juvenile mouse brain in the subacute phase. This suggests that neural autophagy inhibition might be a potential therapeutic target for preventing irradiation-induced late-onset sequelae in childhood brain tumor survivors after radiotherapy.

## Ethics Statement

All experiments were approved by the animal research ethics committee (Gothenburg Committee of the Swedish Agricultural Agency) in accordance with national animal welfare legislation (112-2014).

## Author Contributions

YW, KZ, TL, and YX performed the experiments, analyzed the data, and wrote the manuscript. CX, YS, JR, SZ, and JS performed the experiments and analyzed the data. XW and CZ analyzed the data. CZ, KB, and XW designed the study and revised the manuscript. All of the authors read and approved the final manuscript.

## Conflict of Interest Statement

The authors declare that the research was conducted in the absence of any commercial or financial relationships that could be construed as a potential conflict of interest. The handling Editor is currently editing co-organizing a Research Topic with one of the authors CZ, and confirms the absence of any other collaboration.

## Abbreviations

*Atg7*: autophagy 7
BBB: blood–brain barrier
Cldn11: claudin-11
CNP: 2′,3′-cyclic-nucleotide 3′-phosphodiesterase
DCX: doublecortin
EGL: external germinal layer
GFAP: glial fibrillary acidic protein
IGL: internal granular layer
KO: knockout
MBP: myelin basic protein
ML: molecular layer
MMP-9: matrix metalloproteinase-9
Olig2: oligodendrocyte transcription factor
OPC: oligodendrocyte progenitor cell
PDGFRα: platelet derived growth factor receptor α
WT: wild-type.
